# How Does the Psychological Impact of COVID-19 Affect the Management Strategies of Individuals with Type 1 and Type 2 Diabetes? A Mixed-Method Study

**DOI:** 10.3390/healthcare12171710

**Published:** 2024-08-27

**Authors:** Norah Abdullah Bazek Madkhali

**Affiliations:** Department of Nursing, College of Nursing and Health Sciences, Jazan University, Jazan 45142, Saudi Arabia; namadkhali@jazanu.edu.sa

**Keywords:** diabetic, anxiety, depression, insomnia, healthcare services, self-care-management, COVID-19 pandemic, Saudi Arabia

## Abstract

(1) Background: During and after the pandemic, individuals with type 1 and type 2 diabetes struggled to maintain a healthy lifestyle due to psychological distress and the struggle to accommodate contextual challenges and changes in their family and work obligations and expectations. This study aims to explore the long-term impacts of the pandemic on proactive self-management behaviors and outcomes that consider contextual and environmental factors, such as family and work dynamics. (2) Methods: In this mixed-method study, data were collected from 418 participants using the Hospital Anxiety and Depression Scale (HADS) and the Insomnia Severity Index (ISI), followed by 16 individual interviews. (3) Results: The prevalence of depression was 37.1%, that of anxiety was 59.1%, and that of insomnia was 66.3%. Significant differences were observed in anxiety by age (*p* = 0.02), while individuals with other comorbidities were more likely to report insomnia (*p* = 0.3). Overall, various challenges during the pandemic have exacerbated emotional distress and complicated self-care routines and adherence to healthy lifestyles. (5) Conclusions: The COVID-19 pandemic has prompted individuals with type 1 and 2 diabetes to adopt alternative health-management methods, such as self-care, proactive initiatives, and daily challenges. Enhancing proactiveness, awareness, and an understanding of individuals’ needs is crucial for alleviating stress, controlling disease, and preparing for potential future health crises in the wake of the pandemic’s long-term effects.

## 1. Introduction

Individuals with diabetes experience debilitating psychological symptoms that can negatively impact their mental health [1,2]. There is a dearth of literature concerning individuals‘ experiences with diabetes during and after the pandemic regarding proactive self-care management strategies and contextual challenges that include environmental factors in developing countries, including the Kingdom of Saudi Arabia (KSA). Throughout the pandemic, individuals with type 1 and type 2 diabetes encountered disruptions in healthcare services, changes in proactive self-care management strategies, increased stress and anxiety, limited access to medications and supplies, and reduced social support. Proactive self-care management strategies included telemedicine and remote monitoring, home-based exercise, healthy eating, stress management, medication adherence, and regular monitoring [3].

Various environmental factors, including lockdowns, family dynamics, and work obligations, also influenced the levels of psychological distress among individuals with type 1 and type 2 diabetes during the COVID-19 pandemic. Research suggests that the interplay between these factors may significantly impact the physical and mental well-being of individuals managing diabetes [4]. For example, the impact of lockdowns on self-care management among individuals with diabetes remains controversial. Some studies have found that ongoing social distancing and lockdown measures during the pandemic negatively affect self-care management and the control of blood glucose levels [5,6,7]. However, other studies have demonstrated a positive effect of the lockdown on glycemic control and self-care routines among individuals with diabetes [8,9].

In the KSA, research directly addressing the intersection of COVID-19, Arab culture, family customs, work obligations, and diabetes management is limited. Disruption to family gatherings, the workplace, and social interaction due to COVID-19 restrictions show that the support networks crucial for individuals with type 1 and type 2 diabetes were negatively impacted. Diabetes self-care necessitates a high degree of problem-solving skills, and anxiety and depression both impair these skills. This may result in worse self-care routines and, eventually, worse glycemic management. The current study aims to explore the long-term impacts of the pandemic on proactive self-management behaviors and outcomes that consider contextual and environmental factors, such as family and work dynamics, when assessing and addressing the psychological distress experienced by individuals with type 1 and type 2 diabetes both during and after the pandemic. Further understanding the psychological disturbances that individuals with type 1 and type 2 diabetes might experience is essential to assess and explore the psychological disorders of COVID-19 among individuals with type 1 and type 2 diabetes.

### Aims of the Study

Phase One:

(1) To examine the prevalence of depression, anxiety, and sleep disturbance among individuals with type 1 and type 2 diabetes during the recent COVID-19 pandemic in Jazan, Saudi Arabia.
(2) To evaluate the relationships between depression, anxiety, and insomnia among individuals with type 1 and type 2 diabetes.
(3) To identify the associated factors of depression, anxiety, and insomnia based on demographic characteristics.

Phase Two:

(1) To gain insight into the experiences, during and after the pandemic, of individuals with both type 1 and type 2 diabetes who survived COVID-19 and to identify the strategies and resources used in managing both type 1 and type 2 diabetes.
(2) To explore the contextual challenges and identify the barriers that might hinder individuals with type 1 and type 2 diabetes from prioritizing self-care management and following a healthy lifestyle during and after the pandemic.

## 2. Materials and Methods

### 2.1. Study Design, Setting, and Participants

This study utilized a mixed-method design to investigate the prevalence of psychological distress and its contributing factors among individuals with type 1 and type 2 diabetes. Additionally, it explored the self-care management strategies and contextual challenges experienced by diabetic COVID-19 survivors. This study used a mixed-method segregated design with sequential synthesis, incorporating quantitative (Phase I) and qualitative (Phase II) approaches. This methodology was used to comprehensively explore the experiences of individuals with type 1 and type 2 diabetes during COVID-19 to provide a holistic understanding that accommodates complementary or divergent views and experiences.

**Figure 1:**
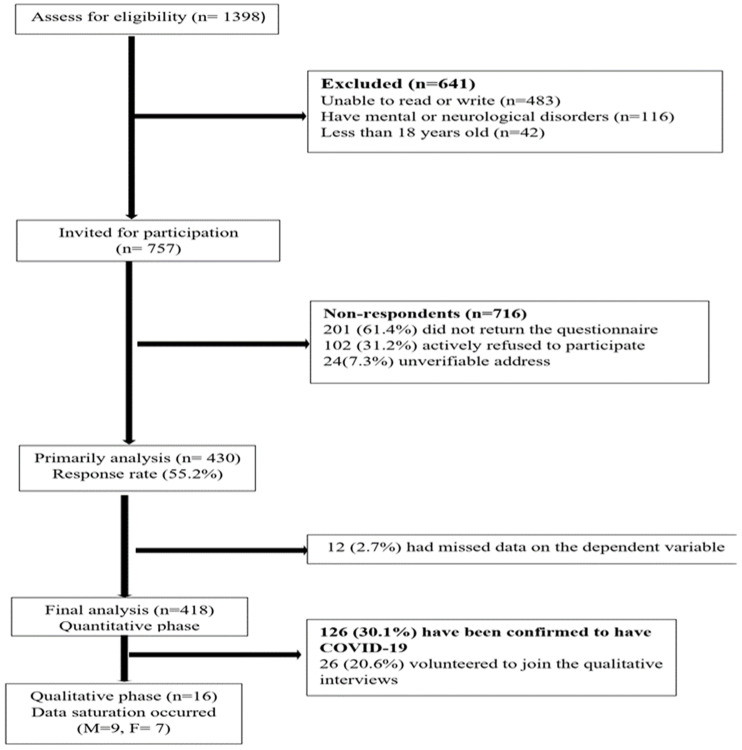
Flow diagram of the recruitment of the participants.

The participants were recruited from chronic disease clinics at primary healthcare centers across the Jazan region. For the quantitative phase, the sample size was determined using the Raosoft sample size calculator (Raosoft, Inc., Seattle, WA, USA) [10], with a confidence level of 95%, a margin of error of 5%, and a target population of 377 required for the findings to be statistically acceptable (as per the population of individuals with type 1 and type 2 diabetes). The data were carefully evaluated along with the eligibility criteria, with 418 potential participants included in this study, as detailed in Figure 1.

Based on the eligibility criteria, the quantitative phase was conducted from Feb 2022 to July 2022. The inclusion criteria for participation in the study were individuals with type 1 or type 2 diabetes over 18 years of age who could speak and write in Arabic. They must have had no known psychiatric or neurological disorders that could interfere with the study participation. In addition, they must have been diagnosed with type 1 or type 2 diabetes more than three years prior. A total of 418 valid questionnaires were received through the online survey. Project nurses and nursing students were made responsible for data collection in the quantitative phase. Before data collection, they took part in a two-day workshop conducted by the principal investigator to orient them to the study’s aim and objectives, including how to use mapping instruments and conduct standardized interviews. Quantitative data collection involved registering information from each patient’s medical record, including demographics and background history. Standardized tools were then administered based on their preferred mode, either as a paper form or as an online survey, and they were given adequate time to complete it. A total of 418 participants completed the survey (Figure 1).

For the qualitative phase, semi-structured interviews were conducted from August 2022 to March 2023. Participants were recruited from the survey study based on meeting the screening criteria for the qualitative phase, which included confirmation of COVID-19 infection. In the initial sample of the quantitative phase (Figure 1), 126 participants were confirmed to have COVID-19, and only 26 volunteered to join the qualitative study. Data saturation was reached after 16 participants were interviewed [11], and the interview times ranged from 30 to 75 min. All interviews were conducted and recorded in Arabic by principal investigators in the patient’s native language and etymology, thereby having similar community and cultural knowledge, which enhanced the research’s credibility [12].

### 2.2. Measurements

#### 2.2.1. Phase I

##### Quantitative Phase

The quantitative phase employed a large-scale cross-sectional design. The survey was developed using an online platform (Qualtrics^®^)and distributed among individuals with type 1 and type 2 at primary healthcare centers across the Jazan region. The questionnaires included detailed demographics, background history, and psychometric scales, including the Arabic versions of the Hospital Anxiety and Depression Scale (HADS) and the Insomnia Severity Index (ISI).

(a) *Demographics*:

Information about the participants’ demographics included gender, age, marital status, educational level, and employment status. The survey also obtained background histories containing the type of diabetes, the time since diagnosis, and other comorbidities. In addition, participants were asked the following two questions: (a) Have you been confirmed to have COVID-19? (b) Have you received the COVID-19 vaccines?

(b) *Depression and Anxiety*:

HADS contains 14 items assessing anxiety (seven items) and depression (seven items), which are rated using a four-point Likert scale (from 0 to 3). The scores in each subscale were computed by summing the corresponding items, with maximum scores of 21 for each subscale [13]. The Arabic version of HADS has good internal consistency (Cronbach’s α = 0.83) [14].

(c) *Insomnia*:

The ISI is a seven-item, self-reported questionnaire assessing insomnia’s nature, severity, and impact [15]. A five-point Likert scale was used to rate each item from 0 = no problem to 4 = very severe problem, yielding a total score ranging from 0 to 28. A previous study reported acceptable psychometric properties for the Arabic version (Cronbach’s α = 0.82) [16].

#### 2.2.2. Phase II

##### Qualitative Phase

An individual semi-structured interview guide that reflects the aims and objectives of the study was developed from the results of the quantitative phase and the literature review (Appendix A). Individual semi-structured interviews explored the participants’ experiences of COVID-19 and the healthcare service they received. The interview guide contains the following areas: (1) the experiences of proactive self-management, including diet and sleeping patterns, physical activity, medication and follow-up, and COVID-19 vaccination; (2) contextual challenges, identifying the barriers and challenges sufferers might face that hinder them from prioritizing proactive self-care management during and after the pandemic. All interviews followed the same guidelines and contained the same prompts, but there were differences due to the dynamics of individual conversations.

##### Data Analysis

In the quantitative phase, the data were entered into SPSS Version 25. Descriptive statistics were used to identify the demographic characteristics of the subjects, including the clinical variables. The Pearson correlation coefficient was applied to determine the relationship between depression, anxiety, and insomnia among subjects. Chi-square and Fisher’s exact tests were employed to identify the association between predictor and outcome variables. Logistic regressions were performed by computing the crude odds ratio (OR) and adjusted odds ratio (aOR) with a 95% confidence interval (CI) to identify the associated factors of depression, anxiety, and insomnia among subjects with COVID-19. The logistic regression variable-selection method was used to identify the best predictors for the backward outcome variables. A *p*-value of <0.05 was considered statistically significant. Gender, age, marital status, level of education, employment status, time since diagnosis, presence of comorbidity, confirmed COVID-19 and vaccine status were included in the full model to run the backward logistic regression analysis for depression, anxiety, and insomnia.

For the qualitative phase, the dataset was analyzed using inductive thematic analysis [17]. This process first involves inductive coding to identify and establish patterns and categories. This is followed by deductive data analysis, looking backward at the data from the themes to determine whether further evidence could be gathered from the transcript to support each theme [18]. Finally, the themes and sub-themes were checked to ensure that they told a straightforward story and captured the depth and breadth of the data. Data analysis and interpretation were cross-verified with a third party who was bilingual in Arabic and English, to maintain the credibility of the data.

## 3. Results

### 3.1. Quantitative Analysis

#### 3.1.1. Demographic Characteristics

A total of 418 valid questionnaires were received through the online survey. Many participants were female (59.3%, *n* = 248) and married (56.5%, *n* = 236). The largest age group was 18 to 30 years (41.1%, *n* = 172), followed by 41 to 50 years (20.1%, *n* = 84). Most participants had obtained a degree (46.2%, *n* = 193), followed by secondary education (36.1%, *n* = 151). Most participants were unemployed (53.8%, *n* = 225), while 30.6% (*n* = 128) were employed. The type 1 diabetes prevalence was higher, at 51.2% (*n* = 214), compared to one-third of the subjects diagnosed with type 2, at 33.3% (*n* = 139). A total of 59.8% (*n* = 250) had only diabetes, and around one-third of the participants had confirmed COVID-19 (30.1%, *n* = 126), with most of them (65.6%, *n* = 274) receiving two doses of a COVID-19 vaccine.

#### 3.1.2. Clinical Variables

Overall, the prevalence of depression was 37.1% (*n* = 155), that of anxiety was 59.1% (*n* = 247), and that of insomnia was 66.3% (*n* = 277). There were no differences in reporting depression, anxiety, and insomnia regarding marital status (*p* = 0.26), educational level (*p* = 0.55), confirmed COVID-19 (*p* = 043), and vaccine status (*p* ˃ 0.94), as detailed in Table 1.

There were significant differences in reports of depression in terms of gender (*p* < 0.05), with the highest proportion of depression among male participants (49% vs. 35.7%) compared with the non-depression groups. Depression differed significantly with regard to employee status, with retired participants being more likely to have depression (23.9% vs. 10.6%).

There were significant differences in anxiety by age (*p* = 0.02), with the highest proportion of anxiety found among participants aged between 18 and 30 (46.2% vs. 33.9%) and among those aged between 41 and 50 (21.1% vs. 18.7%) compared with the non-anxiety group. The insomnia groups had significantly more female participants (62.8% vs. 52.5%), as did those with type 1 diabetes (53.4% vs. 46.8%) and other diabetic groups (17.3% vs. 12.1%) (*p* = 0.04) compared to the non-insomnia groups. The time since diagnosis was significantly correlated with insomnia (M = 9.51, SD = 8.98). Participants with hypertension/heart failure, asthma, diabetes/heart diseases, more than one comorbidity, and other comorbidities were more likely to report insomnia (*p* = 0.03) than the non-insomnia group (Table 1).

#### 3.1.3. Correlation between Depression, Anxiety, and Insomnia

Pearson correlation coefficients were used (Table 2) to assess the associations between depression, anxiety, and insomnia, which were statistically significant (*p* < 0.001).

#### 3.1.4. Predictive Factors Associated with Depression, Anxiety, and Insomnia

Multivariate logistic regressions were conducted to identify the predictors of depression, anxiety, and insomnia (Appendix B). The logistic regression model for depression showed that individuals with anxiety were significantly predicted to suffer from depression (*p* = 0.00). The logistic regression model for anxiety revealed that participants with depression and insomnia are likely to suffer from anxiety (*p* < 0.05). In the logistic regression model for insomnia, the models showed that participants having anxiety and diabetes only were significantly likely to suffer from insomnia (*p* = 0.04)

### 3.2. Qualitative Analysis

**Figure 2:**
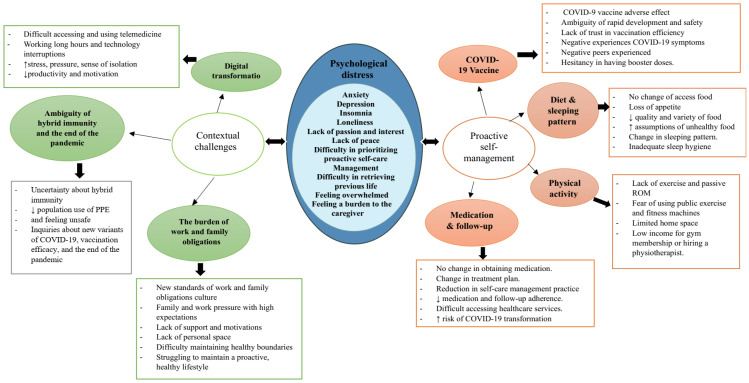
Psychological distress, contextual challenges, and proactive self-management experiences among individuals with diabetes during and after the COVID-19 pandemic.

The thematic analysis of the sixteen semi-structured individual interviews led to the identification of two overarching themes and seven interpretative themes (Figure 2) based on the experiences of individuals with type 1 and type 2 diabetes regarding their proactive self-management and contextual challenges, identifying the issues and needs they face in their social and work contexts and environments during and after the pandemic (Table 3).

#### 3.2.1. Proactive Self-Management

This overarching theme entails the disruption of self-care management strategies and healthy lifestyles related to diet, exercise, medication, and COVID-19 vaccination during and after the pandemic. This theme highlights the challenges faced by participants in regaining their healthy habits.

##### Diet and Sleeping Pattern

This interpretive theme focuses on the impact of COVID-19 and its consequences on changes in diet habits and sleep patterns during and after the pandemic. It explores how these changes negatively affect the control of blood glucose levels and weight. Some participants reported that although they had access to fresh food, they lost appetite and experienced a decreased likelihood of adhering to their regular diet due to feelings of loneliness. Others expressed how the lockdown and its consequences negatively impacted the quality and variety of their regular meals. Older participants mentioned that these changes to their diet were indicative of their emotional state, perceiving themselves as a burden to their family members. In addition, many participants discussed how their daily routines and sleep patterns were disrupted. They mentioned that stress or boredom led them to eat between meals, and their sleep hygiene was inadequate. They described how their sleep habits, including timing, duration, continuity, and alertness or sleepiness, were disrupted at home and in their social environment. They noted that these changes were reflected in elevated blood glucose levels and weight gain.

##### Physical Activity

This interpretative theme highlights the participants’ physical activity changes. For example, vulnerable participants who previously had visits from physiotherapists at home stopped exercising. They shared how they struggled to incorporate self-exercise into their daily lives, which negatively impacted their physical and psychological well-being. Some participants, particularly mothers, had to adjust their physical exercise in their home environment due to the challenge of being constantly surrounded by family members during lockdowns and school closures. They felt distressed and overwhelmed as they could not find time alone at home to maintain their self-care management strategies, including exercising and meditation. Others were concerned about using outdoor gyms and fitness facilities. Due to anxiety, limited home space, and low income, participants found it challenging to return to their everyday routines and engage in regular daily exercise and meditation.

##### Medication and Follow-Up

This interpretative theme highlights participants’ struggles in adhering to their medication and attending follow-up appointments. Some participants feared going to medical institutions due to the perceived risk of infection. They also mentioned not adhering to follow-up appointments, believing it was unnecessary during the pandemic, as they could obtain their regular prescribed medication from pharmacists. Older participants talked about relying on their family members as they were vulnerable and unable to monitor their blood glucose levels and administer insulin injections. They reported a decline in physical and psychological well-being due to the pandemic lockdown and its circumstances. Some participants emphasized how COVID-19 had adversely affected their physical and mental health, leading to fluctuations in their blood glucose levels and modifications to their treatment plans, including insulin injections. Others viewed COVID-19 as a frightening and unknown pandemic, discussing its impact on healthcare system delivery, with a lack of system capacity, increased infection risk, and pressure on healthcare providers. They felt that COVID-19 had adversely affected their healthcare services and communication with healthcare providers.

##### COVID-19 Vaccination

This interpretative theme focuses on COVID-19 vaccination and explores participants’ experiences and attitudes toward the vaccine. Some participants expressed uncertainty and fear about getting vaccinated, citing concerns about the types of vaccines available and the lack of knowledge regarding their short- and long-term effects. Others had misconceptions about COVID-19 and vaccination efficacy. They acknowledged the lifesaving potential of vaccinations and their role in ending the pandemic. Some participants’ views regarding vaccination and the need for booster doses of COVID-19 vaccination were shaped by their personal experiences of vaccination and infection. Many participants doubted the effectiveness of the COVID-19 vaccine in reducing the severity of symptoms and complications. They also perceived the vaccine as unsafe for individuals with compromised immune systems, such as those with diabetes. Additionally, they shared anecdotes of peers experiencing cardiovascular and thrombosis complications after vaccination, which contributed to their hesitancy and fears about receiving booster COVID-19 vaccination doses.

#### 3.2.2. Contextual Challenges to Self-Care Management Strategies

The second overarching theme identifies contextual barriers and hindrances sufferers might face in maintaining a healthy lifestyle during and after the pandemic.

##### Digital Transformation

This interpretive theme discusses the digital transformation and its impact on participants’ lives during and after the pandemic. It focuses on how digital transformation has influenced their family and work responsibilities, medical care, and daily routines. For instance, some older participants expressed challenges in accessing healthcare services through telemedicine due to hearing, vision, and language issues and a lack of knowledge, confidence, and interest in virtual visits with healthcare providers. They also emphasized that digital transformation has heightened feelings of loneliness and isolation. Other participants, especially mothers and employees, believed that the surge in digitalization during and after the pandemic negatively impacted the prioritization of their self-care and ability to manage their responsibilities effectively. They discussed how the digital transformation came with considerable pressure and expectations. They were constrained by work and family obligations and struggled with the interruption of endless emails and messages. They believed that digital transformation generates more demand and creates more significant psychological distress and anxiety in the home environment, which are obstacles to controlling their emotions, self-care management strategies, healthy lifestyle habits, and blood glucose levels. On the other hand, some participants appreciated the innovations in social media and health applications. They found value in using health and self-management tools, such as free fitness and meditation apps. However, they struggled to incorporate these tools into their routines due to family and work commitments and the stressful home environment.

##### The Ambiguity Surrounding Hybrid Immunity and the End of the Pandemic

This interpretive theme explores participants’ uncertainty and concerns about hybrid immunity and perceptions of the end of the pandemic; for example, some participants expressed uncertainty about hybrid immunity against COVID-19 and its ability to protect them from infection, reinfection, and severe COVID-19 symptoms. They shared their experiences of losing loved ones with diabetes to COVID-19 despite receiving three doses of the COVID-19 vaccine. They believed individuals with diabetes were vulnerable and had low immunity and that a combination of SARS-CoV-2 infection and vaccination could not provide immunity against COVID-19 and its variants. Others voiced concerns about family and community members not adhering to personal protective equipment guidelines. They also discussed their uncertainties questioning hybrid immunity and COVID-19 vaccinations. They highlighted that when the general population is lax about personal protective measures, new variants of COVID-19 continue to emerge. Their apprehensions and uncertainties about the future of the pandemic were evident in their expressions of insecurity and ambiguity.

##### The Burden of Work and Family Responsibilities during and after the Pandemic

This interpretive theme discusses how the pandemic and its consequences disrupted the participants’ lives, goals, and plans and how they felt overwhelmed by COVID-19 and its impacts on their health, emotions, and everyday lives. They highlighted the new work environment and standards in family life that emerged during and after the pandemic, expressing negative feelings such as depression, a lack of passion, and a loss of interest in life. They also emphasized the stress caused by COVID-19 and the lack of support and motivation to prioritize self-care management strategies and healthy lifestyles. Some participants mentioned struggling to return to their pre-pandemic habits and adapting to the new standard of life that emerged. They believed that COVID-19 had impacted negatively on their mental health and the self-care management strategies that promote their well-being. Many struggled to find peace given the uncertainty of the post-COVID-19 future. Participants also shared their experiences of disruption and lifestyle transformations, expressing frustration and constraints due to COVID-19’s impact on their home and work environment. They faced challenges in setting and maintaining healthy boundaries in relationships with family members and work colleagues, finding personal space, managing interruptions, and dealing with high family expectations during and after the pandemic. Participants believed that the consequences of COVID-19 have shaped expectations surrounding a ‘new normal’ for family and work obligations, leading to enhanced psychological distress and hindering their ability to maintain a healthy lifestyle and control metrics, such as body weight and blood glucose levels.

## 4. Discussion

The present study explored the psychological impact of COVID-19 on diabetic patient survivors. A mixed-method study was used to identify the prevalence of psychological disturbance among participants and gain deeper insights through semi-structured individual interviews with a subset of participants with confirmed COVID-19. The interviews allowed participants to express their views, interpretations, and experiences of COVID-19. This allowed for the collection of participants’ interpretations and points of view regarding their experiences of psychological disturbances during lockdowns and identified the strategies and resources used in managing their care. It also explored the contextual challenges hindering participant self-management during and after the pandemic.

The Phase I quantitative analysis revealed that the prevalence of depression was 37.1% (*n* = 155), that of anxiety was 59.1% (*n* = 247), and that of insomnia was 66.3%. This finding is consistent with a previous cross-sectional study that found that the prevalence rates of depression and anxiety symptoms in individuals with diabetes living in the Arab Gulf region (*n* = 586) were 61% and 45%, respectively [19]. Symptoms of major depression increased from 11.9% before COVID-19 to 21.3% afterward [20]. A systematic review of 37 studies found that anxiety reached 23% (95% CI = 19–28) in T1DM and 20% (6–40) in T2DM. Meanwhile, diabetes distress was 41% (95% CI = 24–60) for T1DM and 36% for T2DM. The prevalence of stress in T1DM subjects was 79% (95% CI = 49–98). Psychiatric comorbidity and psychosocial issues that impair disease management make diabetics more vulnerable during pandemics and crises [21]. This study shows that there were significant differences in reports of depression in terms of gender (*p* = 0.008), with the highest proportion of depression among female participants (51% vs. 64.3%) compared with the non-depression groups. These results aligned with a previous quantitative study that reported that depression and anxiety were higher in females compared to males [22]. For example, females had a 1.12 times higher risk of depression than males (OR  =  1.12, 95% CI: 0.99 to 1.25, *p*  < 0.001) [23]. This study shows that there were significant differences in anxiety by age (*p* < 0.02), with the highest proportion of anxiety found among participants aged between 18 and 30 (46.2% vs. 33.9%) and among those between 41 and 50 (21.1% vs. 18.7%) compared with the non-anxiety group. However, Parlapani et al. [24] reported that a higher level of anxiety was found in the older age group. Thus, indicating factors for anxiety, such as age, among individuals with diabetes during COVID-19 remain controversial.

This study found that insomnia groups had significantly more female participants (62.8% vs. 52.5%), as did those with type 1 diabetics (53.4% vs. 46.8%) and other diabetic groups (17.3% vs. 12.1%) (*p* = 0.04) compared to the non-insomnia groups. The results reporting the prevalence of insomnia in the current study were relatively high compared with the earlier report from Qatar [25], where insomnia was lower (*n* = 103; 35.8%). This study’s results also showed that participants with hypertension/heart failure, asthma, diabetes/heart diseases, more than one comorbidity, and other comorbidities were more likely to report insomnia (*p* = 0.03) than the non-insomnia group. This finding is consistent with that of Al Meqbali, who showed that participants with heart disease and many comorbidities had a higher risk of insomnia (χ^2^[1,*n* = 922] = 65.57, *p* < 0.001) compared to non-insomnia groups [26]. This study showed a significant correlation between depression, anxiety, and insomnia (*p* < 0.001). During the COVID-19 pandemic, multiple studies highlighted concerns about psychological distress and its impact on sleep pattern disturbances in individuals with diabetes [26,27,28,29]. Therefore, it is crucial to prioritize humanized care, comprehensive care strategies, and coping mechanisms to enhance health and self-care and minimize the psychosocial impacts of the COVID-19 pandemic and future crises or pandemics among this population segment.

This study’s Phase II qualitative findings indicate a high prevalence of anxiety, depression, and insomnia among the participants. These findings provide a deeper insight into the mental health conditions of the participants and their impact on the participants’ proactive self-care management strategies during and after the COVID-19 pandemic. While most participants reported having access to fresh food and their regular diet, they lost their appetite and were less likely to adhere to it due to loneliness. These results provide new insights into how COVID-19 and its consequences adversely affect the caregiver burden of the older participants, as well as the quality and variety of their regular meals. Some participants also discussed how modifying their daily routine and sleeping patterns caused changes in their eating habits in negative ways and caused fluctuating blood sugar levels during and after the pandemic. These results support the previous studies that found that sleep disturbances [30] and higher levels of anxiety and depression [31] negatively impacted glycemic control among individuals with diabetes. Therefore, changes in dietary habits not only interrupt the metabolic control of individuals with diabetes but also jeopardize their overall health [32].

While this study’s findings refute those of previous studies [33,34], they confirm others [3,7,31,35,36,37] that found that the COVID-19 pandemic caused a reduction in physical activity and exercise among individuals with type 1 and type 2 diabetes. This study adds new information to the international literature by identifying vulnerable older participants who normally had physiotherapists visiting them at home but stopped performing their exercises due to COVID-19 and its consequences. They were unable to incorporate self-exercise into their daily life routine. Furthermore, they felt constrained by a lack of appropriate support and COVID-19 social restrictions, which impacted negatively on their physical and psychological well-being. Yu et al. [38] suggested that exercises at different intensity levels facilitate the recovery of older diabetic subjects and highlighted that they impacted the subjects’ immune function positively, thus helping to prevent infection and improve prognoses of COVID-19. On the other hand, some participants, especially mothers, had to adapt their physical activity at home, which caused them to feel distressed, frustrated, and overwhelmed, as they could not maintain their self-care regime due to the lockdowns and school suspensions. Although the pandemic is officially over [39], some participants still harbor concerns about using outdoor gyms and fitness centers and remain anxious about the lax protective measures adopted by the public as new COVID-19 variants remain in circulation. Despite striving for a healthy lifestyle and regular exercise, participants still struggle to prioritize actions for controlling blood glucose and weight. They remain anxious, have limited home space, and have low income, which are crucial factors during and after the COVID-19 pandemic. This study emphasized how COVID-19 and its trajectory adversely affected physical and psychological health, including increased psychological burden, fluctuating blood glucose levels, treatment plan changes, and insulin injection prescriptions. A systematic review of fourteen studies that aimed to discuss the impact of COVID-19 on healthcare utilization globally among individuals with diabetes demonstrated reduced healthcare utilization and adherence to routine healthcare services [40]. While most participants did not experience any change in access to medication and blood glucose control equipment, they confirmed the underutilization of healthcare. However, older participants discussed how COVID-19 restrictions and social isolation hindered their self-care management strategies, including monitoring their blood glucose levels, administering insulin injections, and maintaining foot care. They expressed how they depended on their family members and reported a decline in physical and psychological well-being due to the pandemic lockdown and its circumstances. This study’s findings also offered a further explanation that identified the reasons for underutilization and poor follow-up adherence among individuals with type 1 and type 2 diabetes. These include fear of infection, exposure to unnecessary risk, and perceiving follow-ups as unnecessary, as the participants did not experience any change in obtaining regular prescribed medication from pharmacists. Participants also illustrated the impact of COVID-19 on healthcare system delivery, such as a lack of system capacity and enhanced infection risk and pressure on HCPs. They felt the pandemic adversely affected their healthcare services and communication with HCPs.

While around one-third of the participants in this study (29.4%) received the first dose and two-thirds (65.6%) received the second booster dose of the COVID-19 vaccination, they were open to discussing their hesitancy to have booster doses of the COVID-19 vaccination. They raised inquiries about the ambiguity of the rapid development of COVID-19 vaccinations compared to traditional vaccinations. They also highlighted their negative experiences of COVID-19, although they were vaccinated, and negative peer experiences of having stroke and thrombosis complications. However, this side effect is still controversial, and the incidence rate of severe cardiovascular and neurological disorders is relatively low, most of which are curable or reversible [41,42]. Therefore, the advantage of vaccination outweighs the risk of COVID-19 infection, especially among individuals with chronic illnesses [41,43].

Several studies [44,45,46] highlighted that immunity induced solely by immunizations provides less protection than hybrid immunity induced by infection and vaccinations. However, this study offers further insights into and interpretations of the contextual challenges that cause additional burdens and overwhelming negative feelings during and after the pandemic. For example, many participants raised inquiries and expressed their uncertainty about hybrid immunity and the need for booster doses of the COVID-19 vaccination. They were uncertain how the hybrid immunity and vaccinations would provide more protection and decrease COVID-19 complications when most populations were lenient in using self-proactive equipment and new variants of COVID-19 were still being developed, in addition to COVID-19 vaccines being linked to a slight increase in cardiac and neurovascular disorders. Thus, it is imperative to enhance awareness, vaccination knowledge, and the availability of accurate information to clarify their inquiries and improve vaccination behaviors among individuals with diabetes [47].

The digital transformation significantly contributes to aiding individuals in daily decisions on medications and symptom management, facilitating healthcare coordination, and promoting healthy behavior changes [48,49,50]. This study extends beyond this and explains the lived experiences and interpretations in managing diabetic mellitus and the use of technology during and after COVID-19. Some participants expressed gratitude for the innovation in social media and health applications, like free fitness and meditation applications, which they found beneficial for health and self-care management. However, they struggled to use these applications due to family and work obligations and stressful home environments. For example, mothers and employee participants noted that the rise in digitalization during and after the pandemic negatively impacted their self-care management strategies. They mentioned feeling pressured, overwhelmed by work and family commitments, and disrupted by constant emails and messages. They believed that the digital transformation adds to psychological distress, making it challenging to maintain emotional control, self-care management strategies, healthy habits, and blood glucose levels. It has been shown that women bore greater responsibilities for household chores during the pandemic. Women who work from home are often expected to manage both their professional responsibilities and domestic duties, thus taking on the roles of both working professionals and housewives. This indicates that women are required to perform household and civic labor, further adding to their already significant workload [51]. Thus, employed female participants bore a double burden and struggled to prioritize their self-care management strategies and control their blood glucose levels and emotional distress due to the lack of support and motivation. On the other hand, older participants encountered difficulties with telemedicine due to hearing, vision, and language issues and a lack of knowledge, self-efficacy, and interest in virtual visits. They also felt that the digital transformation increased feelings of loneliness and isolation. Therefore, the negative aspects of digital transformation on the daily experiences of individuals with diabetes should be acknowledged and utilized to build appropriate interventions and offer digital solutions to achieve optimal health outcomes.

This study adds a novel perspective by articulating how the pandemic disrupted participants’ lives, goals, and plans, causing overwhelming feelings about its impact on health and emotions. The participants discussed the new work and family culture during and after the pandemic, expressing negative emotions like depression and a lack of interest in life. They also emphasized COVID-19-related stress and a lack of support and motivation for prioritizing self-management and healthy lifestyles. The participants conveyed their challenges in returning to pre-pandemic habits and adapting to the new post-pandemic lifestyle. They expressed the negative impact of COVID-19 on mental health, highlighting their use of self-care management strategies for health promotion. They also articulated difficulties in finding peace amid the uncertainty of COVID-19’s future. The participants shared their experiences of disruption and lifestyle changes due to COVID-19. They expressed frustration and constraints in home and work environments, struggling with setting healthy boundaries with family and colleagues, managing interruptions, and dealing with high expectations. They believed that the consequences of COVID-19 shaped new norms in family and work obligations, contributing to psychological distress and hindering efforts to maintain a healthy lifestyle and control blood glucose levels. Thus, it is significant to implement manageable, understandable, and meaningful actions to address psychosocial strain and disruptions in daily life and foster a general sense of coping despite challenges [52].

The present study on the self-management of individuals with type 1 and type 2 diabetes during the COVID-19 pandemic was conducted only in the Jizan region, which may limit the generalization of this study due to social and cultural diversity. This research used self-reporting questionnaires for psychological problem assessment, which may differ from clinical diagnostic interviews. The author’s epistemological position may impact their interpretation of participants during data collection and analysis. To address this, the author categorized different research reflexivity levels, including epistemological and personal [53,54]. Future studies should gather information from family members, caregivers, and healthcare providers to better understand challenges related to the self-management of individuals with type 1 and type diabetes.

## 5. Conclusions

The prevalence of depression among individuals with type 1 and type 2 diabetes was 37.1%, that of anxiety was 59.1%, and that of insomnia was 66.3%. No differences were found in the reporting of depression, anxiety, or insomnia based on marital status, educational level, confirmed COVID-19, or vaccine status. Significant differences were observed in anxiety based on age, while individuals with other comorbidities were more likely to report insomnia. Individuals with type 1 and type 2 diabetes experienced changes in self-management, including diet, sleeping patterns, and physical activity. Some experienced changes in their treatment plan due to fluctuations in blood glucose levels. However, they reported a lack of adherence to follow-up appointments and medication due to a fear of COVID-19. Furthermore, they expressed hesitancy toward COVID-19 booster doses due to uncertainty surrounding the vaccine’s rapid development, safety, side effects, and negative experiences. Contextual challenges like the digital transformation, family and work obligations, and ambiguity surrounding hybrid immunity and the end of the pandemic exacerbated emotional distress and complicated self-care routines among individuals with type 1 and type 2 diabetes. Overall, these individuals struggled to maintain a healthy lifestyle due to changes in family and work obligations. Recognizing individuals with diabetes proactive efforts and daily contextual barriers is essential to support their self-care management and help them adapt to long-term side effects.

## Figures and Tables

**Table 1:** Demographic and clinical variables of participants (*n* = 418).

			Depression (*n* = 155, 37.1%)		Non-Depression (*n* = 263, 62.9%)			Anxiety (*n* = 247, 59.1%)		Non-Anxiety (*n* = 171, 40.9%)			Insomnia (*n* = 277, 66.3%)		Non-Insomnia (*n* = 141, 33.7%)		
	*n*	%	*n*	%	*n*	%	*p*	*n*	%	*n*	%	*p*	*n*	%	*n*	%	*p*
**Gender**							0.008 **					0.13					0.04
Male	170	40.7	76	49	94	35.7		93	37.7	77	45		103	37.2	67	47.5	
Female	248	59.3	79	51	263	64.3		154	62.3	94	55		174	62.8	74	52.5	
**Age**							0.26					0.02 *					0.41
18–30	172	41.1	60	38.7	112	42.6		114	46.2	58	33.9		122	44	50	35.5	
31–40	57	13.6	21	13.5	36	13.7		25	10.1	32	18.7		34	12.3	23	16.3	
41–50	84	20.1	26	16.8	58	22.1		52	21.1	32	18.7		55	19.9	29	20.6	
51–60	57	13.6	25	16.1	32	12.2		30	12.1	27	15.8		34	12.3	23	16.3	
More than 60	48	11.5	23	14.8	25	9.5		26	10.5	22	12.9		32	11.6	16	11.3	
**Marital Status**							0.38					0.06					0.26
Married	236	56.5	94	60.6	142	54		129	52.2	107	62.6		151	54.5	85	60.3	
Single	155	37.1	51	32.9	104	39.5		103	41.7	52	30.4		110	39.7	45	31.9	
Others	27	6.5	10	6.5	17	6.5		15	6.1	12	7		16	5.8	11	7.8	
**Education Level**							0.79					0.24					0.55
Basic	74	17.7	25	16.1	49	18.6		40	16.2	34	19.9		47	17	27	19.1	
Secondary	151	36.1	58	37.4	93	35.4		97	39.3	54	31.6		105	37.9	46	32.6	
Degree	193	46.2	72	46.5	121	46		110	44.5	83	48.5		125	45.1	68	48.2	
**Employment Status**							0.001 **					0.25					0.11
Employed	128	30.6	42	27.1	86	32.7		69	27.9	59	34.5		76	27.4	52	36.9	
Retired	65	15.6	37	23.9	28	10.6		37	15	28	16.4		43	15.5	22	15.6	
Unemployed	225	53.8	76	49	149	56.7		141	57.1	84	49.1		158	57	67	47.5	
**Type of Diabetes**							0.33					0.43					0.04
Type 1	214	51.2	79	51	135	51.3		131	53	83	48.5		148	53.4	66	46.8	
Type 2	139	33.3	47	30.3	92	35		76	30.8	63	36.8		81	29.2	58	41.1	
Others	65	15.6	29	18.7	36	13.7		40	16.2	25	14.6		48	17.3	17	12.1	
**Other Comorbidities**							0.10					0.10					0.03
Hypertensive/Heart failure	96	23	39	25.2	57	21.7		61	24.7	35	20.5		68	24.5	28	19.9	
Others	15	3.6	6	3.9	9	3.4		11	4.5	4	2.3		13	4.7	2	1.4	
Asthma	25	6	12	7.7	13	4.9		18	7.3	7	4.1		18	6.5	7	5	
None (Only Diabetic)	250	59.8	81	52.3	169	64.3		135	54.7	115	67.3		152	54.9	98	69.5	
More than two Comorbidities	32	7.7	17	11	15	5.7		22	8.9	10	5.8		26	9.4	6	4.3	
**Are you Confirmed of COVID-19?**							0.10					0.44					0.43
Yes	126	30.1	54	34.8	72	27.4		78	31.6	48	28.1		87	31.4	39	27.7	
No	292	69.9	101	65.2	191	72.6		169	68.4	123	71.9		190	68.6	102	72.3	
**Have you taken vaccines for COVID-19?**							0.75					0.49					0.94
Yes, one Dose	123	29.4	43	27.7	80	30.4		72	29.1	51	29.8		83	30	40	28.4	
Yes, two doses	274	65.6	103	66.5	171	65		160	64.8	114	66.7		180	65	94	66.7	
No	21	5	9	5.8	12	4.6		15	6.1	6	3.5		14	5.1	7	5	

* *p* < 0.05; ** *p* < 0.001.

**Table 2:** Relationships between depression, anxiety, and insomnia.

	Depression	Anxiety	Insomnia
Depression	1	0.579 **	0.452 **
Anxiety		1	0.461 **
Insomnia			1

** *p* < 0.001.

**Table 3:** Thematic analysis and participants quotations of semi-structure interviews (*n* = 16).

Overarching Theme	Interpretative Theme	Participants’ Quotes
**Proactive self-management**	**Diet and sleeping pattern**	*“Thank Allah we have plenty of food, but I could not eat that much. I was alone, and I lost my appetite. I used to eat with my son and grandchildren; they lived close to me next door. After this pandemic, we could not be together. As usual, they brought food to my room. It is too lonely and sad. (P3, F)”*
		*“ I am old and have other comorbidities, so I have a restricted diet. We used to eat with my daughter and grandchildren; after this pandemic, she could not do that as before. She does what she can but has become busy with her children, their distance learning, and her work. The quality of my food has changed. I eat less and do not enjoy my food anymore… I do not want to put an extra burden on her. (P5, M)”*
		*“ I am a teacher, and I used to work with children and have a good daily routine. I worked from home after the lockdown and school suspension, and my daily routine changed. I felt depressed and became irritable and anxious. Being at home 24 h is horrible. I started to eat more. My diet is not as healthy as before. I sleep fewer hours and gain more weight. I could not control myself, and my blood sugar level was fluctuating. (P7, F)”*
		*“I could not retrieve my normal sleeping pattern and peace; the home environment became more noisy and crowded, had more tensions, negative news, stress and tasks or obligations, and not enough personal space, you know, disruption in our daily lives and the lockdown..etc. I could not sleep enough or sleep fewer hours, having more sleeping disruptions and becoming a night person, being exhausted all of the time, and it is difficult to control my blood glucose level, weight, and my eating patterns as well. (P9, M)”*
	**Physical activity**	*“I am an old man, and one physiotherapist used to come to me daily. My kids hired him after my right foot was amputated as I had gangrene. I cannot perform the exercise without help; my muscles are too weak. He helped me to perform the exercise.*
		*“After this pandemic and lockdown, he could no longer work for me as he has worked at the hospital. He might have transmitted the virus to me. I try to perform exercises without help but fail; it is painful, and I feel frustrated. My son helps me during the bath, but I have not performed exercise for a long time. I feel better with exercise. I become more worried, stressed, and irritable since I stopped exercising. I have muscle and bone pain and feel helpless and upset. (P12, M)”*
		*“I used to perform the exercise at home when my children were at school and my husband was at work. I have a daily routine of doing yoga, performing exercises, having online Zumba classes, taking care of myself, and having peace out of everything. After COVID-19, lockdown, and school suspension, I cannot do that anymore; I cannot find peace and a good time. I have tried to perform exercises, but they do not let me do what I want. It is annoying that they are becoming demanding, asking, and disrupting. I have five children and a husband at home; there is much tension and stress. I cannot be alone and have time for myself as before. I cannot control my blood sugar, and I gain more weight. All daily routines are disrupted, including physical activity, diet, and sleeping patterns. I feel depressed and stressed. (M10, F)”*
		*“I used to go to the beach, taking advantage of the public exercise machines, breathing fresh air, and enjoying the view. After this pandemic, it is too difficult. The lockdown has started, and it is not allowed to go out. In addition, it is not safe, and I cannot use the machines there. It is unsterilized. It is for public use. I could not touch or use them anymore. I lived in a small apartment and did not have enough space to exercise. I also have limited income and cannot pay for exercise machines. I feel chained between the room walls and depressed. I did not perform an exercise, and my blood sugar is not controlled. After three years, I felt stuck and could not retrieve my normal life. (P2, M)”*
	**Medication and follow-up**	*“I try not to go to the GP as much as possible; I do not want to be with others; they might have COVID-19, so it is better to be home. My daughter collects my monthly medicine and brings it to me. I have my medication as usual; there is no change. I take my pills every day before breakfast and before dinner. I do not think it needs to be changed. (P15, M)”*
		*“My youngest daughter used to check my blood sugar before having insulin injections, but she cannot visit me and do that regularly due to the lockdown and the COVID-19 consequences. I have tremors and cataracts in my left eye. I cannot do blood sugar tests for myself. I remember not taking my insulin when my blood sugar was low, but I did not know that. Insulin lowers my blood sugar, and I was fainting. My Allah helped me; I could have died at that moment. I feel alone and frustrated. I do not want to die alone or with the wrong insulin dose. This idea makes me more anxious and depressed. My Allah, mercy us. (P16, F)”*
		*“I could not control myself, and my blood sugar level was fluctuating. I was on oral antidiabetic, but recently, the physician prescribed insulin for me. (P13, F)”*
		*“ COVID-19 was horrible and scary. I kept asking about COVID-19 and what was happening, but no one answered. I was sick during the first wave of this pandemic; I went to the hospital with my son. The nurse in the triage room asked me to come back home immediately and not sit on the chair; he told me sorry, COVID-19 patients were everywhere, and we could not protect or serve you right now. He was frustrated and shouting at my son to run away from the hospital. (P11, M)”*
	**COVID-19 vaccination**	*“Many options are available for COVID-19 vaccinations, but which is the best for me? How was it developed so quickly? What are the real risks and side effects? We have heard a lot about its side effects. Some people die after having vaccinations, and some have strokes. What are the side effects in the long term? We do not know the truth about it. (P1, M)”*
		*“I am not sure about COVID-19 vaccination. In the beginning, I was keen to receive vaccination, and I think it is an impressive blessing to save people, get rid of this pandemic, and retrieve our normal life. After having the first dose, I was sick for ten days. Two months later, I was infected and experienced severe symptoms. I think there is no point in having a booster dose. (P4, F).”*
		*“While I had the COVID-19 vaccine, I was infected with COVID-19. I had severe symptoms, and I was hospitalized for a while. Until now, I am still struggling with the long-term effects of chest pain, shortness of breath, fatigue, joint pain..etc. I do not think the COVID-19 vaccine is effective, and I do not think it makes any difference. I think its side effects and complications more than benefits. I know some people had strokes and blood clotting issues after being vaccinated. It is unsuitable for people like us with diabetes. (P13, F)”*
**Contextual challenges to management strategies**	**Digital transformation**	*“ Most medical services and communication channels have moved to the online world. I am an old man who is not good at technology. My daughters tried to teach me, but I could not. I think this makes our life more sophisticated. I could not talk freely online; I felt like I was in prison. I am alone with this mobile. (P5, M)”*
		*“As a mother, this digital technology makes our life horrible, burdens us with more work, and increases the pressure on mothers. I must care for my children’s health and education; distance learning kills me in addition to demanding and depressed husbands, working all the time at home without a break, and tolerating their psychological distress. How could I find time and space to care for myself and look after my health? I am exhausted and cannot control my blood sugar or lifestyle. (P6, F)”*
		*“While digital technology gives us more opportunity to access free and paid online applications such as fitness, exercise, meditation, weight loss, and healthy diet, etc., We could not take advantage of this technology for many reasons, the environmental challenges at home, family and work obligation and unfished online work tasks.”*
	**The ambiguity about hybrid immunity and the end of the pandemic**	*“Everyone talks about hybrid immunity; I do not think this is real for us. My aunt passed away due to COVID-19 when she was 59 years old and had the COVID-19 vaccine three times. She was diabetic like me, and while she survived the first time having a COVID-19 infection, she passed away the second time having this infection. (P14, M)”*
		*“See, around you, nobody is strict with social distancing or wearing masks, even your family members who already know about your medical case, while we hear every time about new variants of COVID-19, how we could have a hybrid community. I do not think this is real. Vaccinations do not work, people are not strict with personal protective measures, and new variants are emerging between time and time, and we feel insecure and safe.”*
	**The burden of work and family responsibilities**	*“I had a healthy lifestyle and quit life. Overnight, we are forced to change our lifestyle and priorities due to COVID-19. Work hard without having a good and clear plan. All our programs were destroyed. Over time, we lose the passion and support to look after ourselves. All the circumstances around me put pressure on me, putting my healthy habits at the bottom of the priorities list. (P1, M)”*
		*“Even now, after lifting precautionary and preventive measures, we still cannot control our lifestyle habits and manage our life patterns to be healthier. I think regaining a lifestyle is not impossible. I used to walk and take advantage of public exercise, but how could I trust it again while we always hear that new variants of COVID have been discovered? We all lost peace somehow in our home and soul (P2, M).”*
		*“During the lockdown, we also lost our boundaries with family members and our space. They became more demanding and complaining and expected a lot from me. This was tough on me as a diabetic patient on Insulin. While the lockdown was over, we all returned to work or school; I could not retrieve my boundaries to have my own space, care for my health, and look after my health as before the pandemic. (P7, F)”*

## Data Availability

No data or materials are publicly available as the participants did not consent to share their interview transcripts or other personal information. Anonymized data may be made available from the corresponding author upon reasonable request.

## Abbreviations

KSA	Kingdom of Saudi Arabia
HADS	Hospital Anxiety and Depression Scale
ISI	Insomnia Severity Index

## Appendix A

**Table A1:** Phase—II (Qualitative study): Semi-structured interview topic guide.

Topic/Themes and Subthemes Covered	Questions Asked
(1) **Proactive self-management**	
(i) **Diet and sleeping pattern:** Understanding how does the COVID-19 pandemic and its trajectory impact diet and sleeping patterns?	₋ How does the COVID-19 pandemic and its trajectory impact your diet and sleeping patterns? ₋ Can you tell me a little more about it? ₋ Could you tell me how you feel about your diet and sleeping pattern?
(ii) **Physical activity** Understanding how the COVID-19 pandemic and its trajectory impact performing exercise and physical activity	₋ How does the COVID-19 pandemic and its trajectory impact your physical activity and performance exercise? ₋ Can you tell me a little more about it? ₋ What challenges do you face that hinder you from performing exercise? ₋ Could you tell me how you feel about your physical activity routine?
(iii) **Medication and follow-up** Understanding how the COVID-19 pandemic and its trajectory impact the utilization of healthcare services and medication adherence.	₋ How does the COVID-19 pandemic and its trajectory impact your medical follow-up and obtaining regular medication? ₋ Can you tell me a little more about it? ₋ Could you tell me how you feel about your treatment plan and medication adherence? ₋ What challenges do you face in this regard?
(iv) **COVID-19 vaccination** Understanding the patient’s experiences and perceptions regarding the COVID-19 vaccinations and booster dose	₋ How do you perceive COVID-19 vaccination? ₋ Can you tell me what comes to mind when you hear about the ‘COVID-19’ vaccine? ……. why? ₋ What does it mean to you…? Can you tell me a little more about it? ₋ Can you tell me more about your experiences with getting the COVID-19 vaccine? How was it? ₋ Could you tell me how you feel about the COVID-19 vaccine? ₋ What do you think about the booster dose?
(2) **Contextual challenges**	
(v) **Digital transformation** Understanding the patient’s digital transformation experiences, including challenges, barriers, and issues.	₋ How do you perceive and experience the innovation in digital transformation during the COVID-19 pandemic? ₋ What do you feel about it? ₋ How do you find telemedicine? ₋ What challenges and barriers do you face and hinder you from using it? ₋ Can you tell me a little more about it?
(vi) **Ambiguity of hybrid immunity and the end of the pandemic** Understanding the patient’s experiences and perceptions about hybrid immunity and the end of the pandemic.	₋ What do you know about hybrid immunity and COVID-19? ₋ Could you tell me about your worries during this time? … how do you feel about it? (situational) … What does it mean to you…? (situational) ₋ What scared you most? ….. and why…? ₋ What do you feel about the end of the pandemic? Do you believe in this? Why? Could you tell me a little more about it?
(vii) **The burden of work and family obligation** Understanding the impact of environmental factors such as work and family obligations on patients’ physical and psychological well-being	₋ How do COVID-19 and its consequences impact your personal life and environment? ₋ What do you feel at that time? ₋ How do family and work obligations impact your self-management strategies? ₋ What do you mean by that (new norms of life and work)? How do you face and experience this change? What do you feel about it? ₋ Why you are struggling? What are your issues and needs?

## Appendix B

**Table A2:** Multivariate logistic regression analyses of factors associated with depression, anxiety, insomnia, and odds ratio (95% CI).

	Depression		Anxiety		Insomnia	
	OR (95% CI)	*p*	OR (95% CI)	*p*	OR (95% CI)	*p*
Other Comorbidities						
None (Only Diabetic)					0.29 (0.08–0.96)	0.04
Depression			0.45 (0.23–0.9)	0.02		
Anxiety	0.47 (0.23–0.94)	0.03			0.31 (0.17–0.56)	0.00
Insomnia			0.31 (0.17–0.58)	0.00		
